# Diagnostic and Therapeutic Advancements in the Field of Animal Reproduction

**DOI:** 10.3390/ani12111457

**Published:** 2022-06-04

**Authors:** Marilena Bazzano, Alessandro Troisi

**Affiliations:** School of Biosciences and Veterinary Medicine, University of Camerino, Via Circonvallazione 93/95, 62024 Matelica, Italy; alessandro.troisi@unicam.it

Reproductive physiology and breeding have fascinated scientist since ancient times, and it is not surprising that explorations in these fields are included among the oldest and most well-documented branches of veterinary medicine [1]. Crop production, farming and the breeding of food-producing animals have driven the growth of civilizations and the need for obtaining food supplies was a great motivation for improvements in animal production and reproduction efficiency [2,3]. At the turn of the last century, advances in reproductive research were mostly driven by the necessity to prevent reproductive diseases and production. In the last few decades, knowledge about animal reproduction has undergone considerable progress, expanding the understandings of pet animals, laboratory species and wildlife conservation and management [4]. In the twenty-first century, research in the field of animal reproduction has not declined. The reliable assessment of reproductive soundness represents one of the main concerns in breeders’ evaluation, as several physiological and pathological conditions may reduce fertility and the possibility of obtaining viable offspring. In our era, research in the field of animal reproduction takes into account the continuous search for innovative diagnostic techniques, such as the application of omics technologies or advanced ultrasonography, to identify specific pathological conditions, as well as the use of novel therapeutic strategies to prevent and handle reproductive diseases, both of which are essential in order to improve breeders’ reproductive efficiency and the health status of their offspring [5]. We have the opportunity to live in an age where several investigative pathways can be adopted to diagnose different reproductive conditions and a number of medical intervention options are available to cope with infertility and increase fertility rates in both pets and food-producing animals. These opportunities were made possible largely thanks to animal reproduction exploration.

Papers accepted for this Special Issue cover distinct aspects of the reproductive sphere. Considering food-producing species, *Kozłowska and collaborators* provided a new ultrasonography-based method to monitor the semen quality of rams and thus maximize fertility by comparing the parameters of basic and advanced semen evaluation tests and testicular blood flow dynamics [6]. 

In the context of new diagnostic supports, serum amyloid A is an acute phase protein that recently gained visibility also in reproduction medicine and two manuscripts of this Special Issue cover the subject. *Wojtysiak and collaborators* provided insights into post-breeding-induced endometritis in the mare, by determining the secretion of anti-inflammatory cytokines and acute-phase proteins in the uterus before and after artificial insemination [7]. As academic editors, we aimed to deepen the knowledge and use of acute phase proteins in daily practice, offering a review article on serum amyloid A [8]. This protein has been widely investigated in pets and food-producing animals as a possible indicator of inflammatory and infective conditions, especially in the field of animal reproduction. With this paper, we provide our contribution in the literature appraisal on the use of serum amyloid A for the diagnosis and monitoring of inflammatory reproductive diseases in animals, critically assessing the usefulness of such markers and summarizing the current state of knowledge. 

The remaining papers cover aspects of canine reproductive medicine. Sub-optimal serum progesterone levels have frequently been linked to pregnancy loss or early parturition, without any scientific evidence to support those claims. In their paper, *Hinderer and collaborators* investigated canine progesterone concentrations during pregnancy and challenged widespread beliefs in the context of hypoluteoidism and progesterone supplementation [9]. Pyometra still represents a major health concern in canine medicine and is considered the most common and important among uterine diseases. As an alternative to the surgical approach, *Rodrigues da Rosa Filho and collaborators* analyzed the effects of two different medical interventions based on aglepristone alone or in combination with prostaglandin in the course of canine pyometra on clinical, laboratory and uterine hemodynamic features [10]. Finally, *Dziecioł and collaborators* presented an observational study on the influence of NSAID drugs on female fertility, monitoring corpora lutea function during the periovulatory phase in the domestic dog [11].

In this Special Issue, we collected contributions by researchers from different countries that provide novel insights about diagnostic approaches for different reproductive disease in domestic species. We are sincerely grateful to all the reviewers who took time to carefully read the submitted manuscripts and provide critical comments which helped to maintain the quality of this issue.
